# Spontaneous Stoma Closure: A Case Report and Review of the Literature

**DOI:** 10.7759/cureus.52403

**Published:** 2024-01-16

**Authors:** Mahdi Albandar, Jumana A Fatani

**Affiliations:** 1 Colorectal Surgery, Minimally Invasive Surgery, Bariatric Surgery, General Surgery, King Saud Medical City, Riyadh, SAU; 2 General Surgery, Specialized Medical Center Hospital, Riyadh, SAU

**Keywords:** cancer, colon, enterocutaneous fistula, retraction, stoma

## Abstract

Stomas serve various purposes, and surgical closure of temporary stomas is typically performed once the underlying reason for their creation has been resolved. However, spontaneous closure of a stoma without surgical intervention is exceptionally rare. Here, we present a case of spontaneous stoma closure. A 67-year-old female presented with symptoms indicative of partial bowel obstruction. A computed tomography (CT) scan revealed circumferential wall thickening involving the transverse, splenic flexure, and proximal descending colon, along with a dilated proximal colon. Urgent intervention led to a total colectomy with ileorectal anastomosis and the creation of a diverting loop ileostomy. Six months later, she was then booked for stoma closure but found that the stoma was already closed, and the patient reported a history of pushing the stoma inside by herself. Spontaneous closure of a stoma is extremely rare. The mechanism behind spontaneous closure is not fully understood. Stoma retraction or the healing process of an enterocutaneous (EC) fistula can be attributable factors. Only six cases of spontaneous stoma closure have been reported in the literature. The technique that has been described by the patient of pushing the stoma inside has not been discussed before. Gradual retraction of the stoma and the healing process of EC fistula are the most common acceptable factors. The technique of pushing the stoma inside could be a helpful factor in spontaneous stoma closure. Long-term follow-up can help in understanding the unclear mechanism of this condition.

## Introduction

An intestinal stoma, created surgically, involves the externalization of either the small bowel or large bowel through the anterior abdominal wall [1]. Stomas are used for various purposes, such as diverting feces to protect a distal anastomosis, relieving an obstruction, or controlling sepsis caused by a perforation [1]. Depending on the intended purpose, a stoma can be temporary or permanent [1]. Surgical closure of temporary stomas is performed once the reason for the stoma has been addressed [1,2]. Spontaneous closure of the stoma without any surgical intervention is very rare [1,3]. We present a case of spontaneous closure of loop ileostomy with a literature review of the spontaneous stoma closure.

## Case presentation

A 67-year-old woman, with a history of hypertension, asthma, and an old stroke, presented with abdominal pain, weight loss, and decreased appetite. She had delayed seeking medical attention and arrived at the emergency room with abdominal distension, frequent vomiting, and an inability to pass stool or flatus. An urgent CT scan revealed a long segment of circumferential wall thickening with marked mucosal hyperenhancement involving the transverse, splenic flexure, and proximal descending colon spanning approximately 20 cm; congested pericolonic vascular mesentery; and proximal dilated colon at risk of perforation. Surgery confirmed a large splenic flexure mass causing proximal dilatation of the colon with evidence of a dissected wall of the cecum. Additionally, there was suspicion of rectosigmoid thickening worrisome of a synchronous tumor. Consequently, a total colectomy with ileorectal anastomosis and a diverting loop ileostomy were performed. Pathology was consistent with infiltrating moderately differentiated adenocarcinoma, with 26 lymph nodes retrieved, and 0/26 were involved by the tumor, staged as pT4N0. Initially, the stoma was draining around 800 mL/24 hours, and the patient was then started on loperamide 8 mg twice daily, at which output was reduced gradually up to 500 mL/24 hours one month after the surgery, with no passage of flatus or stool per rectum.

**Figure 1:**
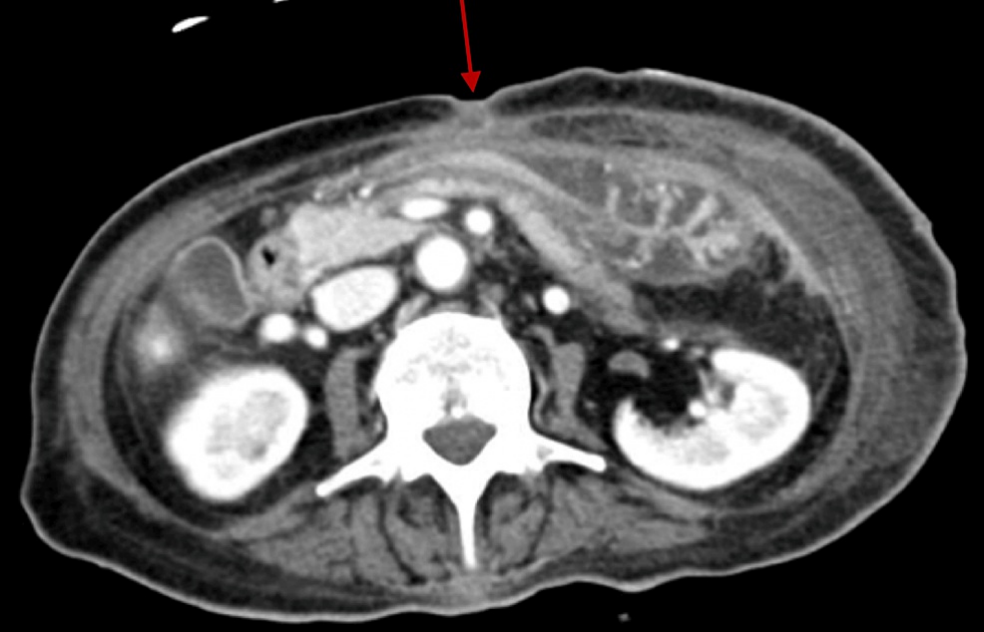
CT scan of the abdomen showing interval closure of the stoma (arrow).

Following chemotherapy and radiotherapy, a subsequent CT scan that displayed interval closure of the stoma (Fig. 1) could not elaborate the details of the stoma opening, thus estimated to be 0 mm, with features suggesting gastrointestinal stromal tumor (GIST) in the gastric body. Upper endoscopy with biopsy was done and showed ulcerated, hemorrhage, purulent, and foreign body giant cell reaction without viable gastric tissue seen for evaluation. A partial gastrectomy was done, which was negative for GIST. She was then booked for stoma closure; upon assessment, the patient reported a normal diet and stool passage per rectum with no stoma output. On examination, a small pinhole-like opening at the stoma site, with no secretion, indicated an auto-stoma closure. A digital rectal examination showed normal stool color with a good tone. The patient mentioned self-manipulation by pushing the protruded stoma inside, eventually experiencing regular stool passage. The patient started pushing the stoma inside manually three months after the surgery. She tried this technique till she was able to push it inside her subcutaneous level, and then she experienced a normal passage of stool per rectum. She started noticing flatus once to twice per day, and gradually the output decreased from the stoma and started passing flatus and stool two months following her manual pushing of the stoma inside. During this time, she did not visit our clinic and started to give local care to the stoma wound till it was closed completely and got granulated six months after surgery. However, the decision was made to explore the stoma from the inside. A diagnostic laparoscopy was performed and revealed a loop of bowel adhesive to the abdominal wall with a completely closed stoma, requiring dissection, resection, and a side-to-side anastomosis. The final pathology of the stroma was negative for any malignancy.

## Discussion

Ileostomies and colostomies are commonly observed [3]. Stoma-related complications can be classified as early (<1 month) or late complications (>1 month) [3]. Early complications include strangulation, obstruction, stenosis, ischemia, dehydration, and mucocutaneous separation fistula. Late complications include retraction, prolapse, parastomal hernia, fistula, variceal bleeding, and ulceration or skin problems [3].

Spontaneous closure of a stoma is extremely rare and usually requires surgical intervention [1,3]. The mechanism behind spontaneous closure is not fully understood, but it can be attributed to stoma retraction or the healing process of an enterocutaneous (EC) fistula [1]. Stoma retraction has an incidence rate of up to 40% in both colostomies and ileostomies [4] and an incidence rate of 20-25% in ileostomies [1,3]. Retraction can occur early in the post-operative period or as a late complication [1]. It involves a gradual separation of the mucocutaneous junction, leading to subcutaneous and intraperitoneal contamination [1]. Factors contributing to retraction include excessive tension on the stoma due to insufficient bowel mobilization, bowel and mesentery edema caused by peritoneal inflammation in emergency situations, mucocutaneous dehiscence from ischemia or stoma necrosis, inadequate approximation of the mucosa to the dermal layer of the skin, peristomal infection, or insufficient maturation due to malnutrition [1]. Other factors, such as high body mass index (BMI), steroid use, malnutrition, diabetes, and smoking, may also play a role in spontaneous stoma retraction [5].

Another potential mechanism for spontaneous closure is the healing process of an EC fistula, as stomas are considered iatrogenically controlled EC fistulae [1-3]. The stoma gradually converts into a lateral fistula through retraction [4]. Roughly one-third of EC fistulae are managed conservatively [1,2]. Stomas typically close through surgical intervention, either via primary repair or resection followed by anastomosis [2,3]. Common causes of EC fistulae include anastomotic breakdown, bowel or mesentery injury, history of radiation therapy, intestinal obstruction, inflammatory bowel disease, mesenteric vascular disease, trauma, or intra-abdominal sepsis [2]. Delayed healing of an EC fistula can be attributed to factors such as foreign bodies, radiation, infection or inflammation, epithelialization, neoplasms, and distal obstruction, known as the "FRIEND" acronym [2,6]. Additional factors include fistulae with a high output stoma (>500 mL/24 hours), involvement of over 50% of the bowel circumference, and a fistula tract length of less than 2.5 cm [2]. Spontaneous closure of EC fistulae can occur if there is no distal obstruction, the bowel is healthy, and the patient is in an anabolic state [1].

To date, only six cases of spontaneous stoma closure have been reported in the literature, with four involving loop ileostomies and two with loop colostomies. Table 1 summarizes the cases that were reported in the literature. Notably, four cases occurred in emergency settings, while two were elective surgeries. Noteworthy is that some patients, including ours, underwent chemotherapy, radiotherapy, or TB treatment, possibly affecting the stoma closure, suggesting a potential role of these treatments in the pathogenesis of spontaneous closure [5]. Most cases displayed gradual stoma retraction with complete retraction of the stoma, which was observed within a range of six weeks to six months, and conservative management was employed in all cases. Our case exhibited complete stoma closure after six months, with self-manipulation by pushing the protruded stoma inside. No stoma retraction was observed during the follow-up. This unique technique self-manipulation has not been published or discussed in the literature.

**Table 1:** Summary of reported cases in the literature

Article	Age	Gender	ER/Elective	Cause	Type of stoma	Closure	Intervention	Follow-up	Medications
Pandit et al., 2016 [1]	64	M	ER	Rectosigmoid perforation	Loop colostomy	Complete closure and epithelialization of the stoma site after 3 months	No	1 year	-
Pandit et al., 2016 [1]	45	M	ER	Rectal adenocarcinoma	Loop ileostomy	Complete retraction of the at 6 weeks, complete epithelialization of the stoma site at 3 months	No	6 months	Adjuvant chemotherapy
Saxena et al., 2015 [2]	26	F	ER	Perforation peritonitis	Loop ileostomy	Complete closure of the stoma after 6 months, complete epithelialization of the stoma site after 8 months	No	-	Anti-TB
Saxena et al., 2022 [3]	18	M	ER	Perforation of hollow viscera	Loop ileostomy	Complete closure after 4 months	No	-	-
Thota et al., 2022 [4]	42	M	Elective	Hirschsprung's disease	Loop colostomy	Complete closure after 8 weeks post-surgery	No	-	-
Alyami et al., 2016 [6]	65	F	Elective	Hereditary non-polyposis colorectal cancer type-I (HNPCC-I)	Loop ileostomy	Complete epithelization 10 weeks after surgery	No	-	Adjuvant chemotherapy

## Conclusions

Spontaneous stoma closure remains a rare occurrence with an unclear mechanism. Factors contributing to this phenomenon include stoma retraction and the healing process of an EC fistula. The novel technique of self-manipulation in closing the stoma might offer insights into understanding this condition. Continued long-term follow-up can help in understanding the unclear mechanism of this condition.
